# Self-Calibration Sensor for Contactless Voltage Measurement Based on Dynamic Capacitance

**DOI:** 10.3390/s23083851

**Published:** 2023-04-10

**Authors:** Chunguang Suo, Rujin Huang, Guoqiong Zhou, Wenbin Zhang, Yanyun Wang, Mingxing He

**Affiliations:** 1 College of Science, Kunming University of Science and Technology, Kunming 650504, China; suochunguang@kust.edu.cn (C.S.); 20212111077@stu.kust.edu.cn (R.H.); wangyanyun@stu.kust.edu.cn (Y.W.); 20212111069@stu.kust.edu.cn (M.H.); 2 College of Mechanical and Electrical Engineering, Kunming University of Science and Technology, Kunming 650504, China; zwbscg@126.com

**Keywords:** noncontact, voltage measurement, dynamic capacitance, self-calibration, voltage sensor

## Abstract

Noncontact voltage measurement has the advantages of simple handling, high construction safety, and not being affected by line insulation. However, in practical measurement of noncontact voltage, sensor gain is affected by wire diameter, wire insulation material, and relative position deviation. At the same time, it is also subject to interference from interphase or peripheral coupling electric fields. This paper proposes a noncontact voltage measurement self-calibration method based on dynamic capacitance, which realizes self-calibration of sensor gain through unknown line voltage to be measured. Firstly, the basic principle of the self-calibration method for noncontact voltage measurement based on dynamic capacitance is introduced. Subsequently, the sensor model and parameters were optimized through error analysis and simulation research. Based on this, a sensor prototype and remote dynamic capacitance control unit that can shield against interference are developed. Finally, the accuracy test, anti-interference ability test, and line adaptability test of the sensor prototype were conducted. The accuracy test showed that the maximum relative error of voltage amplitude was 0.89%, and the phase relative error was 1.57%. The anti-interference ability test showed that the error offset was 0.25% when there were interference sources. The line adaptability test shows that the maximum relative error in testing different types of lines is 1.01%.

## 1. Introduction

Technologies such as power metering, power equipment maintenance, power quality monitoring, and substation area optimization and transformation are continuously advancing with the national level of urban and rural power construction. The sensing and detection of electrical quantities play a pivotal role in the above application scenarios [1,2,3]. Recently, in the field of electrical quantity sensing and detection, there are relatively mature noncontact measurement methods for current, such as using a Roche coil or Hall sensor to measure the magnetic field around the wire to obtain wire current [4,5]. However, in the field of voltage measurement, especially in the 220/380 V low-voltage distribution network due to the lack of mature noncontact voltage measurement methods, when it is necessary to collect voltage data at a certain point, most of them use power outage construction and connect intelligent meters or electric energy information collection terminals on the line to obtain the required key information such as voltage, power, harmonic content, etc. [6,7,8]. This way of requiring power-off installation limits the large area arrangement of voltage sensors in power systems.

The excellent characteristics of noncontact voltage measurement, which does not require electrical connection with the measured object, have made this technology a research hotspot in different fields in recent years. Examples include: the acquisition of weak biological potential [9,10], relay protection [11], online overvoltage monitoring [12,13], and partial discharge monitoring [14,15]. However, contactless voltage measurement has not yet been applied in power systems where known frequency responses are required, due to the uncertainty of sensor gain in field applications of contactless voltage sensors. The gain of a noncontact voltage sensor is affected by the type of wire to be measured, the relative position of the wire, and temperature and humidity. At the same time, it is also subject to interference from interphase or peripheral coupling electric fields. In order to solve the above-mentioned problem of the inability to reconstruct the line voltage due to the difficulty in determining the voltage sensor gain, the researcher has done the following related research.

The author of [16] proposed a voltage measurement method for 10 kV overhead lines using the principle of spatial capacitance voltage division. Based on field applications, the influence of factors such as the laying height of the overhead lines, the ambient temperature and humidity, and the distance from the installation point to the tower on the measurement results was analyzed. The fixed parametric capacitor is welded to the PCB (Process Control Block, PCB) as the primary side capacitance, and the capacitance between PCB and ground is calculated by finite element simulation to determine the sensor gain. However, this method requires that the high-voltage line be directly connected to the parametric capacitor. The author of [17,18] use a predesigned parasitic capacitance value to perform a series of calculations on the original voltage data obtained by coupling, and finally obtains the voltage value of the line to be tested. In this design, there is a problem that the calculated parasitic capacitance value is not equal to the actual parasitic capacitance value. The author of [19,20] uses a known excitation source to achieve system identification to calibrate noncontact voltage sensors. However, this method requires calibration based on known line types, and it is necessary to keep the calibration process consistent with the relative position of the probe and the wire in the actual measurement process. Without special design of the probe structure, it is difficult to achieve this goal. Therefore, currently this method is mostly used for specially designed GIS (Geographic Information System, GIS) tanks to achieve VFTO (Very Fast Transient Overvoltage, VFTO) measurement. The author of [21,22] greatly improves the accuracy of measurement by injecting reference signals to eliminate the influence of unknown capacitance between the probe and the conductor. However, due to the absence of a shielding layer, this method is prone to interference from surrounding coupling electric fields when measuring voltage on both overhead transmission lines and complex low-voltage distribution network lines. The author of [23,24] proposed a D-dot transmission line voltage measurement based on Gaussian integration. Reconstruct the line voltage through the relationship between the electric field value obtained from multiple sensors and the position of the sensor. However, this measurement method is more rigorous for actual measurement scenarios and sensor placement locations, requiring accurate sensor offline and ground distance. However, the exact spatial location of overhead transmission lines is usually unknown and dynamic in practice, and the sagging of the lines, as well as the presence of trees or buildings on the ground, will affect the distribution of electric fields.

Aiming at the uncertainty of the gain of noncontact voltage sensors in practical measurement, this paper proposes a self-calibration method for noncontact voltage measurement based on dynamic capacitance. This method uses an unknown excitation source (line voltage to be measured) to achieve self-calibration of sensor gain in actual measurement scenarios. This article first introduces the basic principle of noncontact voltage measurement self-calibration based on dynamic capacitance, analyzes the problem of sensor gain uncertainty caused by probe wire coupling capacitance uncertainty in noncontact voltage measurement, and proposes a noncontact voltage self-calibration method based on dynamic capacitance. Subsequently, the sensor model and parameters were optimized through error analysis and simulation research. On this basis, an anti-interference sensor prototype and a dynamic capacitance control unit were developed. Finally, the accuracy test, anti-interference ability test, and line adaptability test of the sensor prototype are carried out. The experimental measurement accuracy proves the feasibility of this method. The research results may provide a new direction for the research and development of transmission line voltage measurement methods. Finally, the shortcomings of sensors and future plans are discussed.

## 2. Principle of Voltage Measurement

### 2.1. Noncontact Voltage Measurement Based on Electric Field Coupling

**Figure 1:**
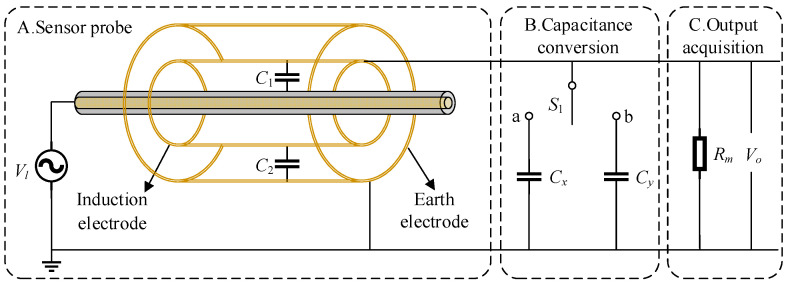
Contactless voltage measurement model based on electric field coupling.

As shown in Figure 1, a traditional electric field coupled voltage measurement sensor consists of part A and part C, including an inner induction electrode and an outer earth electrode. The induction electrode forms a coupling capacitance *C*_1_ with the wire; the induction electrode forms a coupling capacitance *C*_2_ with the earth electrode. The induction electrode is connected to the earth electrode through the sampling resistor *R_m_*, and the ground electrode is connected to the actual ground. If the lead voltage is *V_l_*, and the voltage signal collected across the sampling resistor is *V_o_*, the output voltage of the sensor can be expressed as [20]:

(1) $$V_{o}=\frac{j\omega R_{m}C_{1}}{1+j\omega R_{m}(C_{1}+C_{2})}V_{l}$$

The frequency response of *V_o_* is the frequency response of a high-pass filter with an angular frequency of $f_{1}=2\pi R_{m}\left(C_{1}+C_{2}\right)$. If the operating frequency of the voltage sensor is much greater than $f_{1}$, Equation (1) can be simplified as [20]:

(2) $$V_{o}=\frac{C_{1}}{C_{1}+C_{2}}V_{l}$$

If the coupling capacitance *C*_1_, *C*_2_ and the output voltage *V_o_* can be obtained, the line voltage *V_l_* can be reconstructed using Equation (2). The voltage divider capacitance *C*_2_ can be a fixed structural capacitance value, which can be obtained through high-precision digital bridges. The output voltage *V_o_* can be obtained in real time by means of signal acquisition instruments or acquisition circuits. The size of the coupling capacitance is affected by the wire diameter of the measured wire, the thickness of the insulation layer, and the distance between the probe and the wire. According to $C_{1}=\varepsilon_{0}\varepsilon_{r}S/d$, the dielectric constant of the insulating layer will affect $\varepsilon_{r}$, the wire diameter of the wire will affect the relative area *S*, and the thickness of the insulating layer will affect *d*. These values are unknown in actual measurements, so they cannot be calculated by formula.

**Figure 2:**
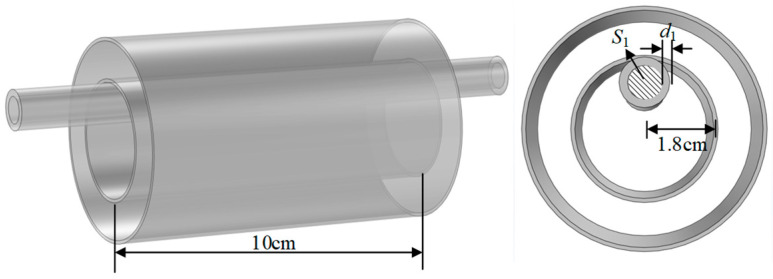
Finite element simulation model and parameters.

In order to quantify the impact of the factors mentioned above on the coupling capacitance, this paper uses the finite element simulation software COMSOL Multiphysics 6.0 to calculate the capacitance values of the wire probe under different types of wires. Considering the placement position of the wire in practical applications, the established model is shown in Figure 2. The inner electrode length of the sensor probe is 10 cm, and the inner electrode radius is 1.8 cm. The cross-sectional area of the wire to be tested is *S*_2_, and the thickness of the insulation layer is *d*_1_. Five kinds of polyvinyl chloride insulated wires in the national standard [25] are selected in the simulation. The calculated coupling capacitance and capacitance change percentage *ε* are shown in Table 1.

According to the simulation results, there is a difference of 14.7% between the line probe capacitance values of 10 mm^2^–1 mm and 16 mm^2^–1 mm, while there is a difference of 37.8% between the line probe capacitance values of 10 mm^2^–1 mm and 70 mm^2^–1.4 mm. As the diameter of the line to be measured increases, the capacitance between the probe and the electrode will increase irregularly. In the above simulation, if we further consider the relative offset position between the probe and the wire, as well as the impact of inconsistent insulating layer media, the resulting capacitance value will have a greater difference. If the voltage is reconstructed by substituting the same capacitance value into Equation (2), the error is proportional to the offset rate of the coupling capacitance.

### 2.2. A Method Based on Dynamic Capacitance Self-Calibration

In order to solve the problem that the line voltage cannot be accurately reconstructed due to the uncertainty of the coupling capacitance *C*_1_ in this paper, a noncontact voltage sensor based on dynamic capacitance is proposed. As shown in Figure 1, on the basis of Part A and Part C, a transformation unit B is added. When the switch is turned to a, the voltage output by the sensor can be expressed as shown in Equation (3). When the switch is turned to b, the voltage output by the sensor can be expressed as shown in Equation (4):

(3) $$V_{ox}=\frac{j\omega R_{m}C_{1}}{1+j\omega R_{m}(C_{1}+C_{2}+C_{x})}V_{l}$$

(4) $$V_{oy}=\frac{j\omega R_{m}C_{1}}{1+j\omega R_{m}(C_{1}+C_{2}+C_{y})}V_{l}$$

The frequency response of $V_{o1}$ and $V_{o2}$ is the frequency response of high-pass filters with angular frequencies $f_{x}=2\pi R_{m}{\left(C_{1}+C_{2}+C_{x}\right)}^{-1}$ and $f_{y}=2\pi R_{m}{\left(C_{1}+C_{2}+C_{y}\right)}^{-1}$, respectively. If the operating frequency of the voltage sensor is much greater than $f_{x}$ and $f_{y}$, Equations (3) and (4) can be simplified as follows:

(5) $$V_{ox}=\frac{C_{1}}{C_{1}+C_{2}+C_{x}}V_{l}$$

(6) $$V_{oy}=\frac{C_{1}}{C_{1}+C_{2}+C_{y}}V_{l}$$

Combining (5) and (6) and eliminating *C*_1_, it can be obtained:

(7) $$V_{l}=\frac{V_{ox}V_{oy}(C_{x}-C_{y})}{V_{x}(C_{2}+C_{x})-V_{y}(C_{2}+C_{y})}$$

### 2.3. Error Analysis and Model Optimization

The selection of capacitance parameters is the key to reducing calibration errors. When there is an error in the sensor output voltage $V_{o}$ in actual measurement, using Equation (7) for calibration may lead to a significant increase in the error. Expanding the denominator terms of Equation (7) can obtain:

(8) $$V_{ox}(C_{x}+C_{2})-V_{oy}(C_{y}+C_{2})=V_{l}C_{1}(\frac{C_{2}+C_{x}}{C_{1}+C_{2}+C_{x}}-\frac{C_{2}+C_{y}}{C_{1}+C_{2}+C_{y}})$$

If $C_{2}+C_{x}$ >> $C_{1}$, $C_{2}+C_{y}$ >> $C_{1}$, then $\frac{C_{x}+C_{2}}{C_{1}+C_{2}+C_{x}}$, $\frac{C_{y}+C_{2}}{C_{1}+C_{2}+C_{y}}$ → 1, $\frac{C_{x}}{C_{1}+C_{2}+C_{x}}-\frac{C_{y}}{C_{1}+C_{2}+C_{y}}$ → 0, $V_{x}(C_{x}+C_{2})-V_{y}(C_{y}+C_{2})$ → 0, which will lead to a significant increase in the error. In order to reduce the propagation of errors, a feasible method is to make $\frac{C_{x}+C_{2}}{C_{1}+C_{2}+C_{x}}$→ 1, while $\frac{C_{y}+C_{2}}{C_{1}+C_{2}+C_{y}}$ tends to zero as much as possible.

**Figure 3:**
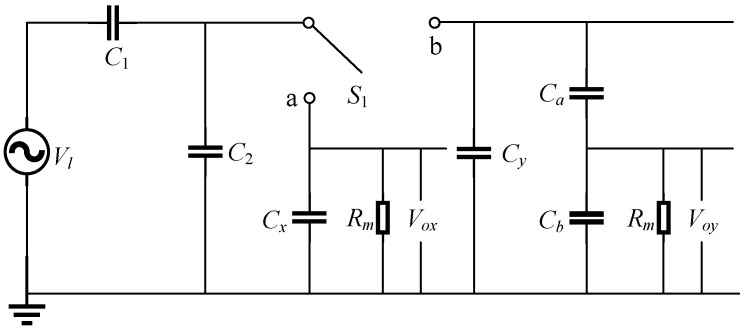
Optimized circuit topology.

However, making the structural capacitance *C*_2_ and the lumped capacitance *C_y_* have the same or similar order of magnitude as the capacitance *C*_1_ will result in a low partial voltage ratio, making it difficult to use a data acquisition instrument or a microcontroller at the output port for direct collection. To maintain high accuracy and improve the voltage divider ratio, Figure 3 shows a design modification that uses two additional capacitors *C_a_* and *C_b_* to set the voltage divider. According to the equivalent circuit diagram, when the switch is turned to *b*, Equation (6) becomes Equation (9), where $k=C_{a}/C_{a}+C_{b}$:

(9) $$V_{oy}=\frac{kC_{1}}{C_{1}+C_{2}+C_{y}+kC_{b}}V_{l}$$

Combining Equations (5) and (9), it can be obtained:

(10) $$V_{l}=\frac{V_{ox}V_{oy}(C_{y}-C_{x}-kC_{b})}{V_{oy}(C_{2}+C_{y}+kC_{b})-kV_{ox}(C_{2}+C_{x})}$$

If necessary, the voltage waveform can be reconstructed using the calculated calibration voltage from Equation (11). When the calibration is completed, the switch is turned to a, and if the relative position of the sensor and the line to be measured is no longer changed, the waveform of the line to be measured can be reconstructed through the real-time waveform $V_{ox}$ output from the sensor and the proportional coefficient *K*, which is expressed as:

(11) $$K=\frac{V_{l}}{V_{ox}}$$

## 3. Design and Application of Self-Calibrating Voltage Sensor

### 3.1. Design and Parameter Selection of Sensor Probe

**Figure 4:**
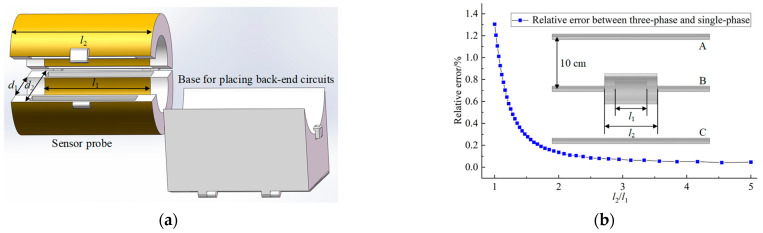
(**a**) Schematic diagram of coaxial induction probe. (**b**) Analysis of the influence of *l*_2_/*l*_1_ on shielding effect.

To facilitate the measurement of line voltage and effectively shield against interphase interference or peripheral electric field interference, the coaxial induction probe designed in this article is shown in Figure 4a. It consists of an induction electrode, an insulating medium, a grounding shielding electrode, an opening and closing hinge, an opening and closing buckle, and a bottom for placing a back-end circuit. The insulating dielectric layer uses a hollow design, using air as the medium to reduce the structural capacitance *C*_2_ so that it has the same or similar order of magnitude as the capacitance *C*_2_. The earth shielding electrode is used to shield external electric field interference, and the hinge is opened and closed to facilitate placement of the wire to be measured in the shaft probe.

As shown in Figure 4b, *l*_1_ and *l*_2_ are the lengths of the inner and outer electrodes, respectively. A design with a better shielding effect is to make *l*_1_ < *l*_2_. Figure 4b shows the analysis results of the impact of using COMSOL finite element simulation on the shielding effect of $l_{2}/l_{1}$ pairs. The percentage difference between the induced voltage output of the probe when applying excitation only to B-phase and simultaneously to phases A, B, and C is plotted as a line, as shown by the blue line segment in the figure. It can be seen that the shielding capability of the coaxial probe is positively correlated with $l_{2}/l_{1}$, but the relative error decreases below 0.2% and continues to increase $l_{2}/l_{1}$, which has little benefit to increasing the shielding. At the same time, the longer *l*_1_ have a larger sensing area, which can capture electric field signals in a larger range, enhancing the load carrying capacity of the back-end circuit. After comprehensive consideration, this article selects $l_{2}/l_{1}$ = 2, and the parameters of the sensor probe are shown in Table 2.

### 3.2. Circuit Topology Parameter Selection

The premise of calibration using Equation (9) is that the sensor works after the inflection point frequency of the high-pass filter, where the output is independent of resistance and frequency. The purpose of this article is to measure the 50 Hz power frequency line voltage; that is, when measuring 50 Hz, the sensor is behind the inflection point frequency of the high pass filter. This requires that the values of $f_{x}=2\pi R_{m}{\left(C_{1}+C_{2}+C_{x}\right)}^{-1}$ < 50, $f_{y}=2\pi R_{m}{\left(C_{1}+C_{2}+C_{y}+kC_{b}\right)}^{-1}$ < 50, which at least meet:

(12) $$R_{m}(C_{1}+C_{2}+C_{x})>3.18\times 10^{-3}$$

(13) $$R_{m}(C_{1}+C_{2}+C_{y}+kC_{b})>3.18\times 10^{-3}$$

To meet the above equation, the selected circuit component parameters are shown in Table 3, and all components are calibrated using digital bridge.

### 3.3. Switch Control and Voltage Calibration Steps

**Figure 5:**
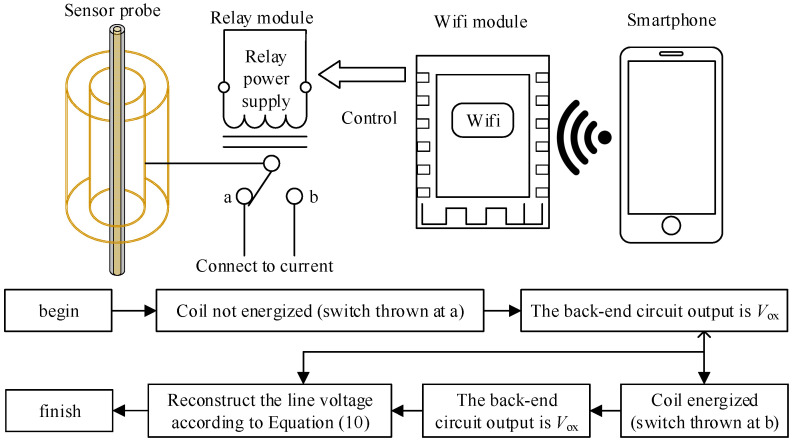
Calibration control elements and process.

The switch used in this article is a relay switch. When it is turned on, its resistance is greater than 1 GΩ, which can be considered as an open circuit. When it is closed, its resistance is less than 75 mΩ. This means that no new stray parameters are introduced during opening and closing. Meanwhile, the smartphone and WiFi module can also be used with diodes and triodes to control its opening and closing. Using the output values of the sensor before and after capacitance conversion, the line voltage can be reconstructed according to Equation (10) to achieve remote calibration of noncontact voltage measurement. The calibration control element and process are shown in Figure 5.

## 4. Experimental Testing and Result Analysis

### 4.1. Establishment of Experimental Platform

**Figure 6:**
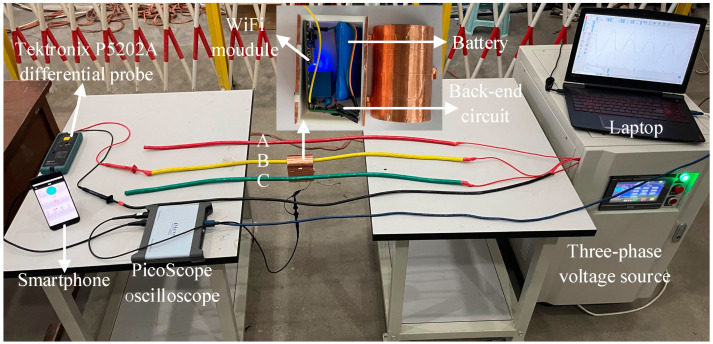
Experimental testing platform.

To verify the feasibility of the dynamic-capacitance-based self-calibration method, an experimental platform was built under laboratory conditions, and on-site measurements were shown in Figure 6. The measured AC voltage is from ANB13-1 KA, which can provide 40 Hz to 100 Hz, 0–300 *V*_rms_ three-phase voltage output. Using a 16-bit resolution PicoScope5000D PC oscilloscope (PicoTech, Cambridgeshire, UK) to collect sensor output, the sampling rate is 62.5 MS/s. The collected signals are connected to a mobile personal computer via USB and displayed through software PicoScope 7.120 (PicoTech, Cambridgeshire, UK). The mobile personal computer is powered and grounded through three plugs. The amplitude and phase of the AC output voltage are calibrated using a Tektronix P5202A differential probe (Tektronix, Inc., Beaverton, OR, USA), and smartphone is used to control switch variation.

During the experiment, the line is passed through the coaxial sense probe, the sense electrode of the probe is connected to the back-end circuit. The back-end circuit is connected to the oscilloscope to display the real-time waveform. A +3.7 V battery and a +3.7 V to ±5 V module are used to power the voltage follower connected to the back-end of $R_{m}$ and the WiFi module. The Tektronix P5202A differential probe is connected to both the B-phase and zero wire terminals.

### 4.2. Amplitude and Phase Accuracy Test

Carry out the test on the established experimental platform, control the three-phase voltage source to output only the B-phase voltage, and perform the following experimental steps. In the first step of testing, use the smartphone control switch to turn to a and record the output of the signal processing circuit observed from the oscilloscope. In the second step of testing, use the smartphone control to turn the switch to b, and record the output of the signal processing circuit observed from the oscilloscope and the actual output voltage measured from the Tektronix P5202A. The output range of the AC source is increased from 100 *V*_rms_ to 300 *V*_rms_ in steps of 10 *V*_rms_. Repeat the above steps and complete the experiment. The recorded results and the voltage reconstructed using Equation (10) are shown in Table 4.

Figure 6 shows the fitting curve and relative error characteristics of the reconstructed voltage and the actual output voltage *ε*; it is expressed as:

(14) $$\varepsilon =\frac{V_{r}-V_{a}}{V_{a}}\times 100\%$$

**Figure 7:**
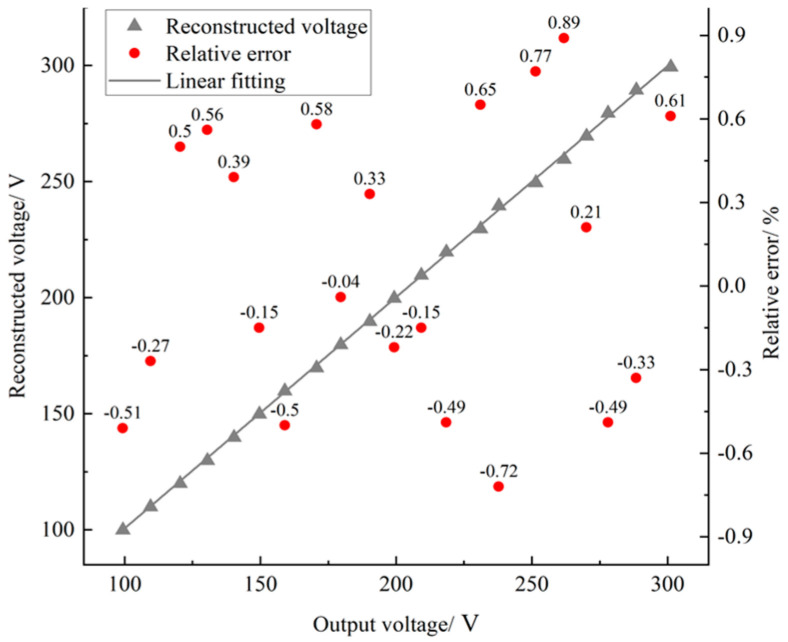
Error characteristics of actual output voltage and reconstructed voltage.

As can be seen from Figure 7, in the test of amplitude accuracy test, the maximum error between the reconstructed voltage and the actual output voltage is –0.89%. The calculation of reconstruction voltage is shown in Equation (10), and the calculation of relative error is shown in Equation (14).

**Figure 8:**
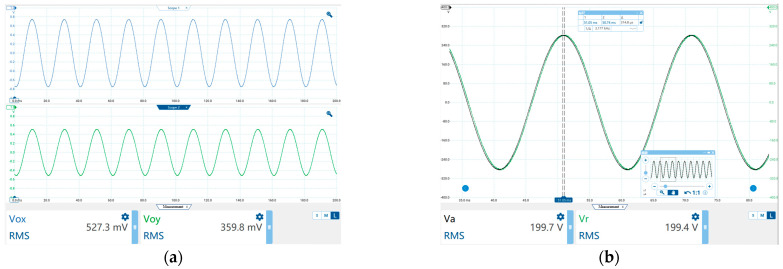
(**a**) When the actual voltage is 200 *V*_rms_, the sensor output before and after the sensor capacitance conversion. (**b**) Comparison between reconstructed voltage and actual voltage.

Figure 8a shows the voltage output waveform of the sensor before and after capacitance conversion when the voltage source output voltage is 200 *V*_rms_. In the experiment, the reconstructed voltage was compared with the measurement results of the Tektronix P5202A differential probe. Figure 8b shows a comparison of the reconstructed signal with the actual signal. The black waveform is the real-time waveform of the Tektronix P5202A differential probe, and the green waveform is the waveform reconstructed using Equation (11). Figure 8b shows that the time difference Δ*t* between two waveforms is 314.8 μs. For a power frequency voltage with a period T of 0.02 s, the relative phase error is Δ*t*/T·100% = 1.57%.

### 4.3. Anti-Interference Ability Test

**Figure 9:**
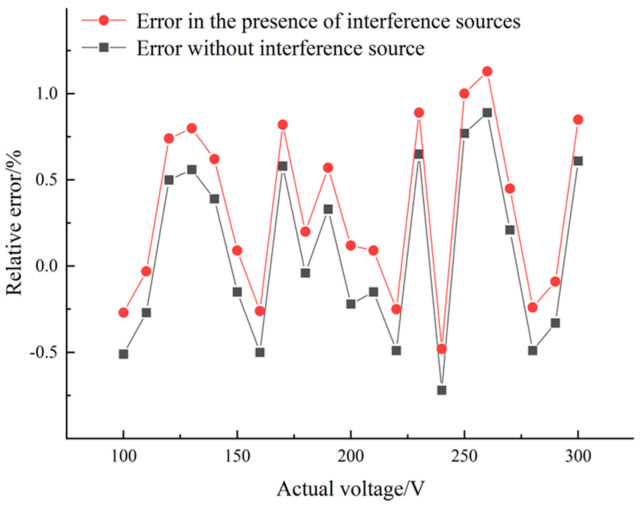
Reconstructed voltage and output voltage error characteristics with and without interference sources.

In order to verify the anti-interference ability of the coaxial probe, the following experimental tests are conducted on the experimental platform shown in Figure 6. Based on the above accuracy measurement experiments, apply the same voltage to the phase-A and phase-C as the phase-B. The voltage source output range increases from 100 $V_{\mathrm{rms}}$ to 300 $V_{\mathrm{rms}}$ in steps of 10 $V_{\mathrm{rms}}$. Consistent with the accuracy testing experimental steps, the recorded output voltage is calculated using Equation (10). The reconstructed voltage and the actual output voltage are calculated using Equation (14) to obtain the relative error with and without interference sources. The error points with and without interference sources are plotted as shown in Figure 9. As can be seen from the figure, compared to the error of reconstructing the voltage without interference sources, the overall error offset with interference sources is 0.25%. This indicates that the coaxial probe has good anti-interference ability and can effectively reduce interphase interference or surrounding coupling electric field interference.

### 4.4. Conductor Adaptability Test

**Figure 10:**
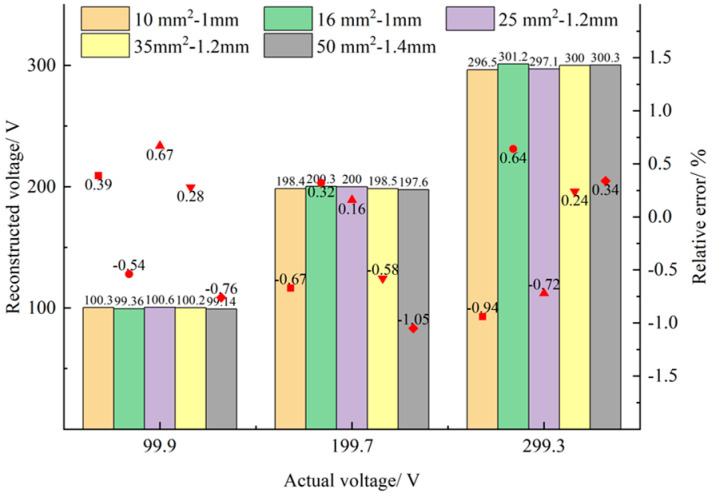
Comparison Diagram of Experiments on Different Types of Circuits.

In order to verify that the proposed self-calibration method can be used for measurement on different target lines, five kinds of polyvinyl chloride insulated wires in the national standard [26] were selected for testing, and their cross-sectional area-insulation layer thickness specifications were 10 ${\mathrm{mm}}^{2}$–1 mm, 16 ${\mathrm{mm}}^{2}$–1 mm, 25 ${\mathrm{mm}}^{2}$–1.2 mm, 35 ${\mathrm{mm}}^{2}$–1.4 mm, and 50 ${\mathrm{mm}}^{2}$–1.4 mm, respectively. Set the output voltage of the AC source to 100 $V_{\mathrm{rms}}$, 200 $V_{\mathrm{rms}}$, and 300 $V_{\mathrm{rms}}$ (the actual output is *Va*), and replace the above types of wires in turn for testing. All wires are randomly placed in the sensing probe without special fixation. The obtained reconstruction voltage and relative error values are shown in Figure 10, the calculation of reconstruction voltage is shown in Equation (10), and the calculation of relative error is shown in Equation (14).

As can be seen from Figure 10, for the five different types of wires tested, the maximum error between the actual output voltage and the reconstructed voltage is –0.94%, 0.64%, –0.72%, –0.58%, and –1.05%, respectively. The experimental results show that the influence of wire diameter on measurement accuracy can be ignored.

## 5. Conclusions

(1) Aiming at the difficulty in determining the sensor gain in practical measurement of traditional capacitive coupled noncontact voltage sensors, a dynamic capacitive noncontact voltage measurement self-calibration method is proposed to achieve self-calibration of the sensor gain in practical measurement.
(2) Theoretical research and transfer function analysis were conducted on the proposed method. Through error analysis and simulation research, the model and parameter optimization design of the dynamic capacitor conversion system were carried out. Based on this, a prototype of anti-interference sensor probe and remote dynamic capacitance control unit were developed.
(3) The calibration accuracy test was conducted using a sensor prototype at a power frequency voltage of 100 *V*_rms_ to 300 *V*_rms_. The results showed that the maximum amplitude error was 0.89%, and the phase error was 1.57%. Subsequently, an anti-interference capability test was conducted, and compared to the error of the reconstructed voltage without interference sources, the overall error offset with interference sources was 0.25%. Finally, adaptability tests were conducted on different types of circuits. The test results show that the maximum relative error is 1.01%, and the measurement line has a small impact on the calibration accuracy.

According to the test results, the noncontact voltage measurement self-calibration sensor based on dynamic capacitance can achieve self-calibration of sensor gain under different measurement scenarios through unknown excitation sources (line voltages to be measured). Using the developed coaxial shielded probe, measurements can be made under strong electromagnetic interference conditions. However, further research is still needed. Currently, only research and testing on the voltage level of low-voltage distribution network lines have been conducted, and further research on higher voltage levels will be carried out in the future. The method in this article is only applicable to voltage measurement in the presence of an effective ground point. In the future, we will study the principle of self-calibration for sensor measurement without hanging a ground wire.

## Figures and Tables

**Table 1:** Simulation Values of Capacitance under Different Specifications of Lines.

*S*_1_/mm^2^	*d*_1_/mm	*C*_1_/pF	Change Rate/%
10	1	9.122	/
16	1	10.69	14.7
25	1.2	11.22	18.7
35	1.2	12.53	27.2
50	1.4	13.07	30.2
70	1.4	14.66	37.8

**Table 2:** Dimensions of coaxial induction probe.

Parameter	Value	Parameter	Value
*l*_1_/cm	4	*l*_2_/cm	8
*d*_1_/cm	3	*d*_2_/cm	5.4

**Table 3:** Calibrate system capacitance and resistance parameter values.

Parameter	Value	Parameter	Value
$C_{2}$/pF	6.46	$C_{x}$/nF	2.07
$C_{y}$/pF	5.08	$C_{a}$/pF	31.1
$C_{b}$/nF	1.96	$R_{m}$/MΩ	20

**Table 4:** Reconstitution accuracy test results.

*V_ox_*/mV	*V_oy_*/mV	*V_r_*/V	*V_a_*/V	*V_ox_*/mV	*V_oy_*/mV	*V_r_*/V	*V_a_*/V
263.6	179.8	99.4	99.9	553.4	377.6	209.3	209.6
289.9	197.8	109.6	109.9	579.7	395.4	218.5	219.6
316.3	216	120.5	119.9	606.2	414	231.1	229.6
342.5	233.9	130.5	129.8	632.7	431.4	237.8	239.5
369.1	252	140.3	139.8	659	450.1	251.4	249.5
395.4	269.8	149.6	149.8	685.4	468.2	261.8	259.5
421.8	287.7	159	159.8	711.7	485.8	270.1	269.5
448.1	306	170.7	169.7	738.1	503.4	278	279.4
474.5	323.8	179.6	179.7	764.5	521.5	288.4	289.4
500.8	341.9	190.3	189.7	789.8	539.4	301.1	299.3
527.3	359.8	199.3	199.7				

## Data Availability

The datasets generated and analyzed during the current study are also available from the corresponding author upon reasonable request.
